# Author Correction: Mutational and putative neoantigen load predict clinical benefit of adoptive T cell therapy in melanoma

**DOI:** 10.1038/s41467-020-15531-2

**Published:** 2020-04-01

**Authors:** Martin Lauss, Marco Donia, Katja Harbst, Rikke Andersen, Shamik Mitra, Frida Rosengren, Maryem Salim, Johan Vallon-Christersson, Therese Törngren, Anders Kvist, Markus Ringnér, Inge Marie Svane, Göran Jönsson

**Affiliations:** 1 Division of Oncology and Pathology, Department of Clinical Sciences Lund, Faculty of Medicine, Lund University, Scheelegatan 2, Medicon Village, 22185 Lund, Sweden; 2 Center for Cancer Immune Therapy, Department of Hematology, Herlev Ringvej 75, 2730 Herlev, Denmark; 3 Department of Oncology, Copenhagen University Hospital Herlev, Herlev Ringvej 75, 2730 Herlev, Denmark; 4 Department of Biology, National Bioinformatics Infrastructure Sweden, Science for Life Laboratory, Lund University, Sölvegatan 35, 22362 Lund, Sweden

**Keywords:** Melanoma, Tumour immunology

Correction to: *Nature Communications* 10.1038/s41467-017-01460-0, published online 23 November 2017.

The original version of this article omitted the following from the Acknowledgements: ‘This project has received funding from the European Union’s Horizon 2020 research and innovation programme under the Marie Skłodowska-Curie grant agreement No 641458’. This has now been corrected in both the PDF and HTML versions of the article.

