# Supplementary material for: Mutational and putative neoantigen load predict clinical benefit of adoptive T cell therapy in melanoma
Source: Nat Commun. 2017 Nov 23;8:1738. doi: 10.1038/s41467-017-01460-0 (PMC5701046; doi:10.1038/s41467-017-01460-0)
Supplement: Supplementary file 1 — Supplementary Information [file 41467_2017_1460_MOESM1_ESM.pdf]

Supplementary Information

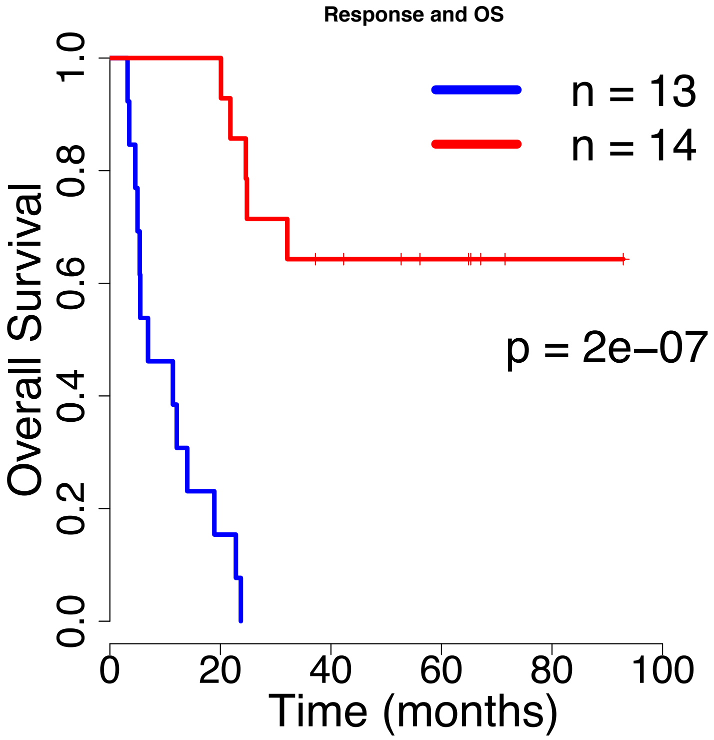

Supplementary Figure 1. Overall survival for the group of clinical benefit (1, red) and no-benefit (0, blue) as defined in the Material & Methods section. P-value from Cox regression.

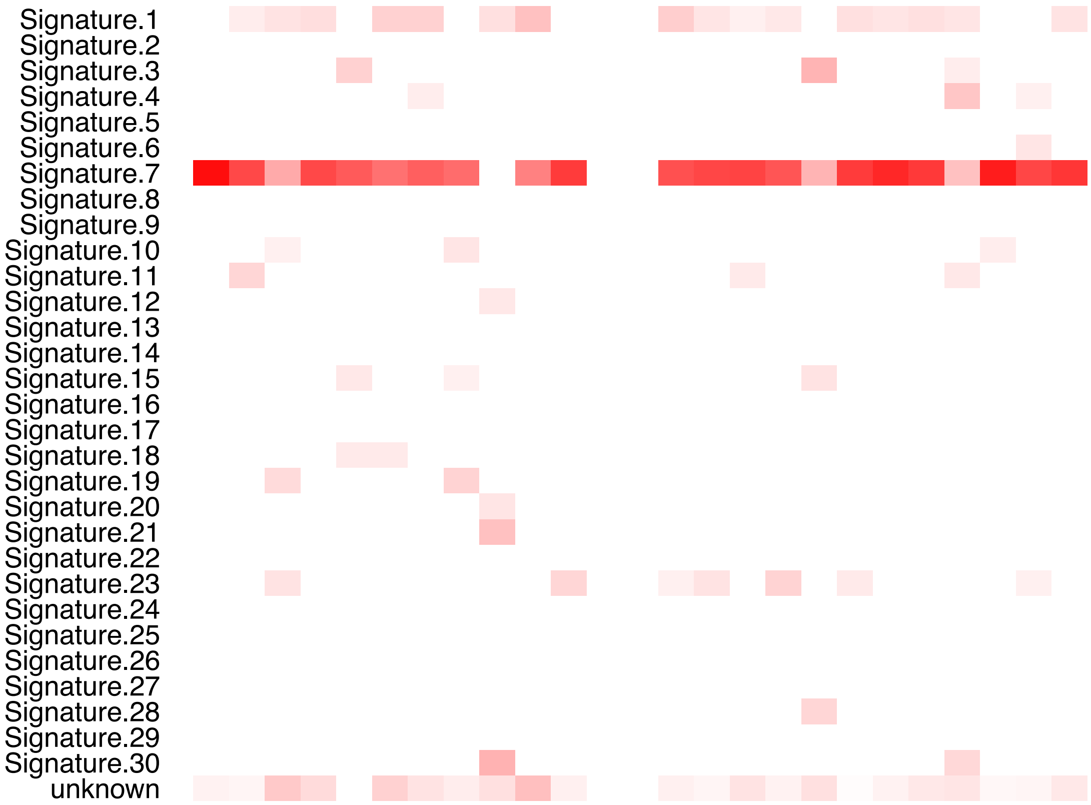

Supplementary Figure 2. Mutation signatures [1] of the pre-ACT tumor samples. Signature 7 mutations have UV-induced DNA damage etiology. On the left is the group of no clinical benefit from ACT, on the right – with clinical benefit.

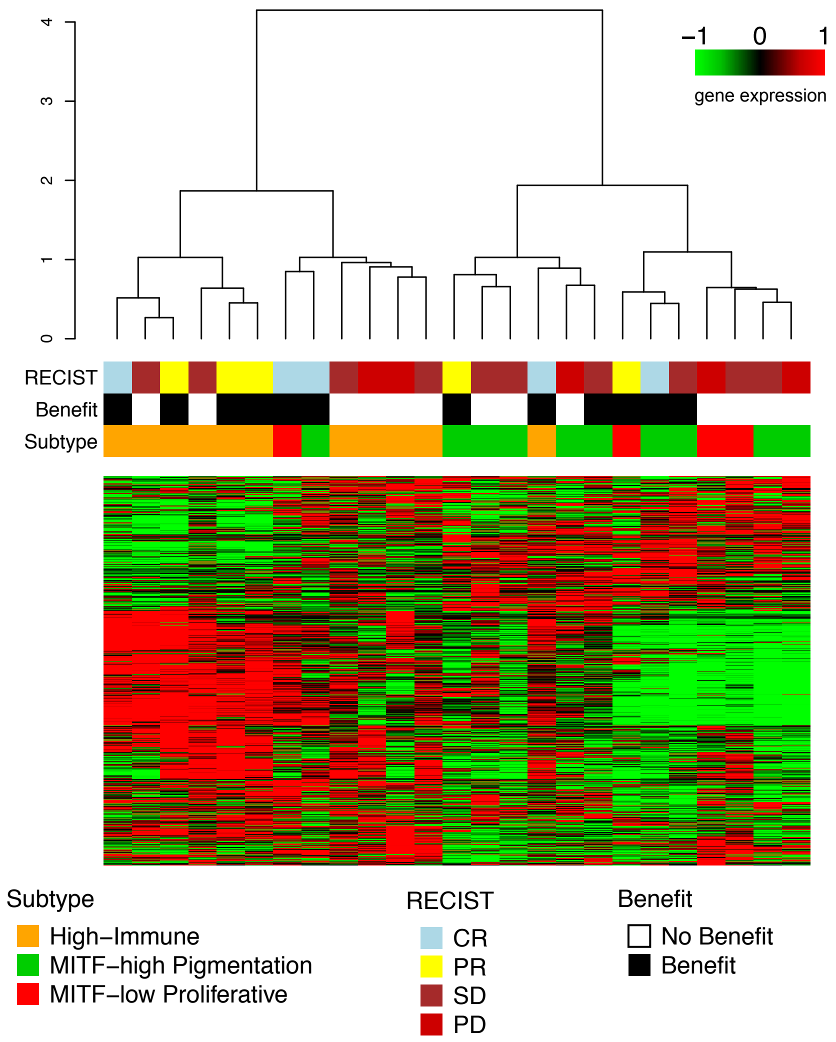

Supplementary Figure 3. Unsupervised clustering of ACT data. Sample distance by Pearson correlation, agglomeration by Ward's algorithm. RECIST, clinical subtypes, and gene expression phenotypes from Jonsson et al[2].

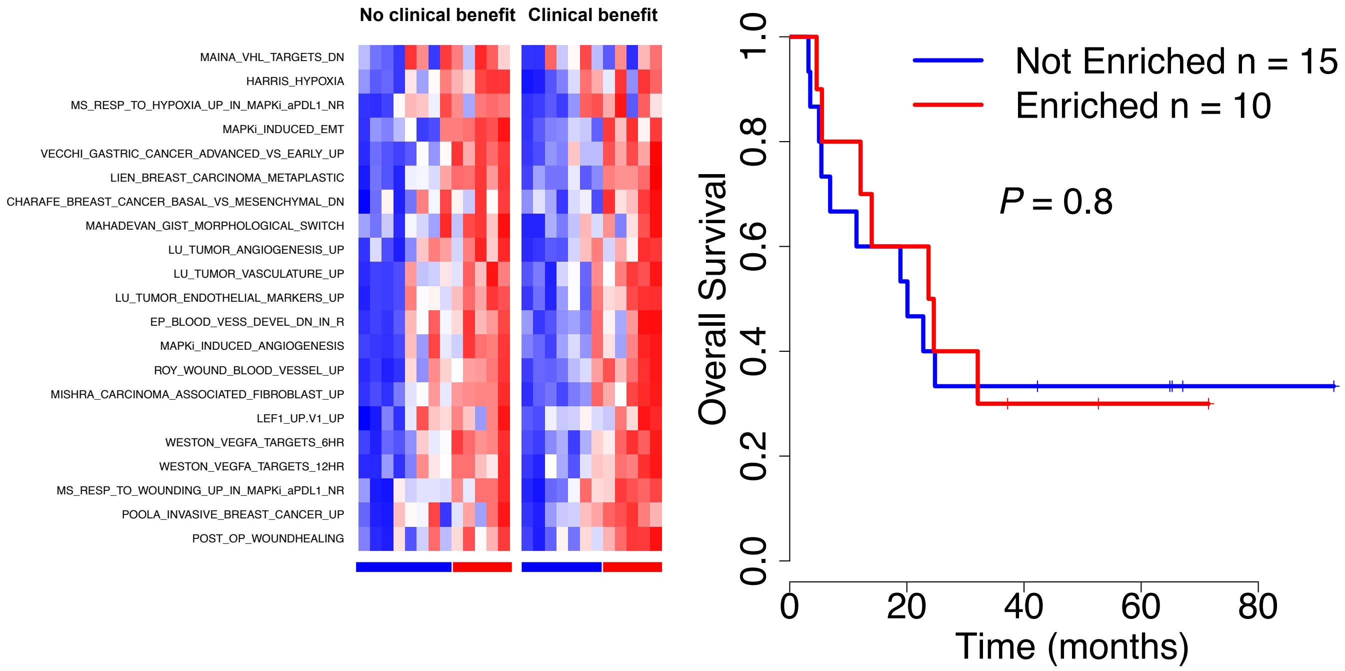

Supplementary Figure 4. IPRES signatures as described by Hugo et al. [3] applied to the ACT cohort. Left panel: Heatmap of z-scores of IPRES signatures. Samples within the response groups are ordered by mean z-scores of GSVA values and samples marked in red have crossed the mean threshold of 0.35 and accordingly were called “IPRES-enriched”. Right panel: Kaplan-Meier plot for overall survival of patients with IPRES-enriched and IPRES-not-enriched samples. P-value from Cox regression.

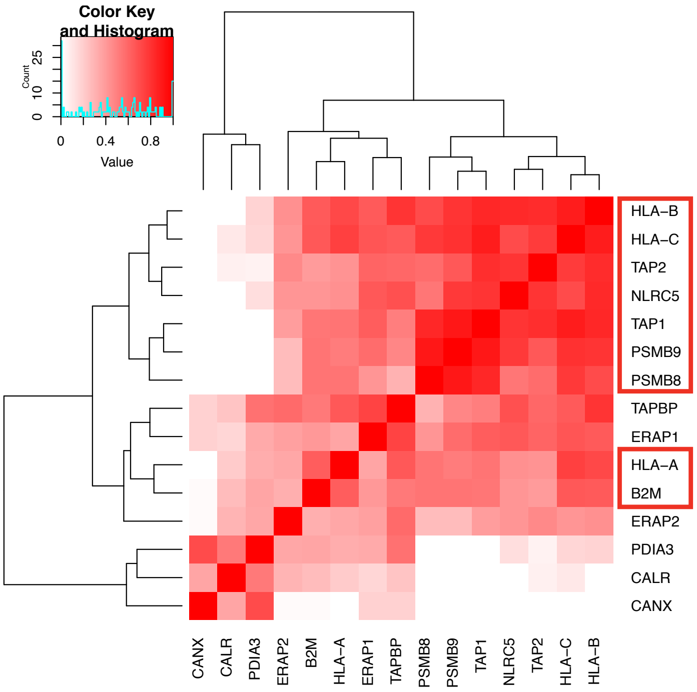

Supplementary Figure 5. Correlation matrix of MHC-I pathway genes in the ACT cohort. Genes in the red boxes displayed highest correlation and thus were termed as “core” MHC-I genes. These genes were used to create the MHC-I score.

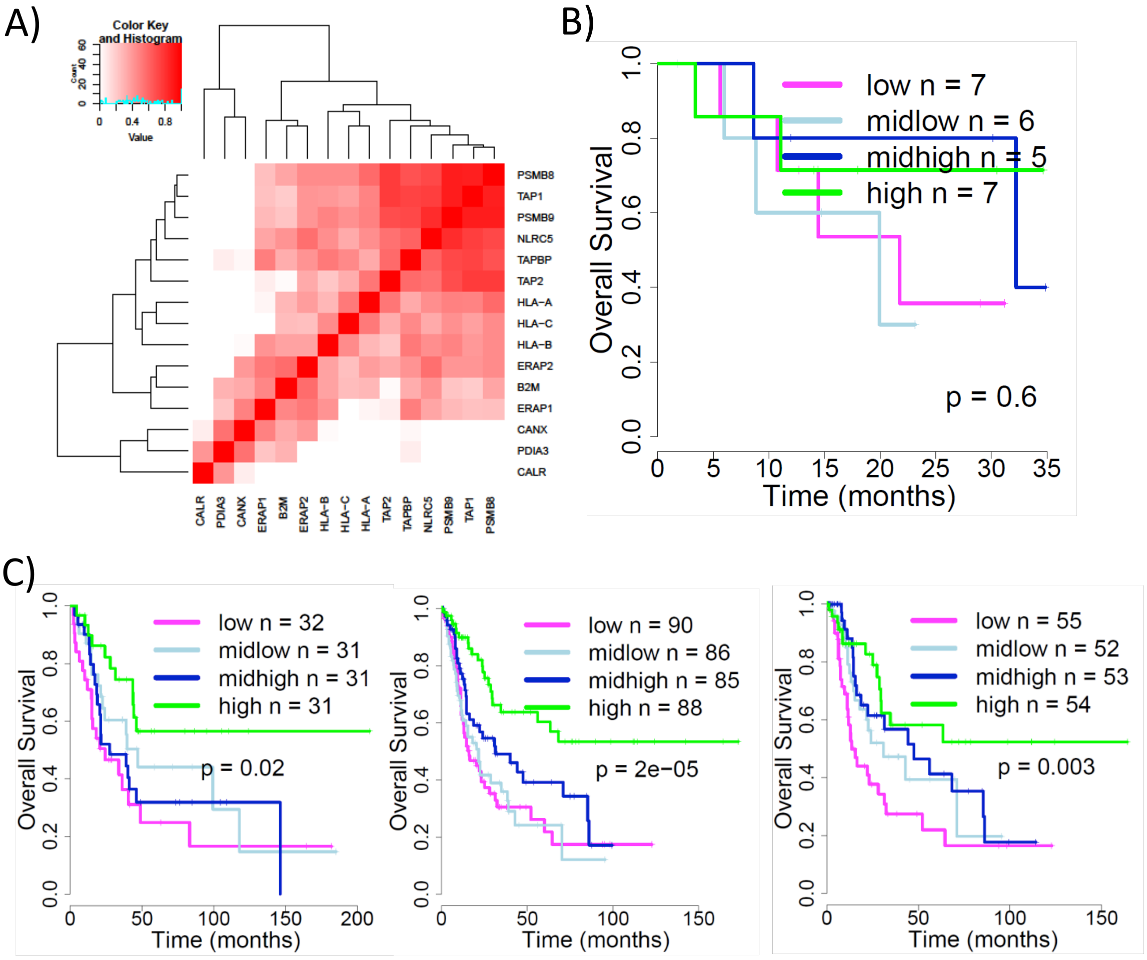

Supplementary Figure 6. Assessment of significance of MHC class I antigen processing and presentation pathway expression in independent data sets **A**) Co-expression of MHC class I pathway genes in Hugo et al. [3]. **B**) Expression of MHC class I pathway genes is not associated with survival following PD-1 inhibition in immunotherapy naïve melanoma patients in the Hugo et al. data [3]. **C)** MHC-I score had prognostic value in the Lund cohort (left) and TCGA cohort[4,5] of all metastases (middle) or regional lymph node metastases (right), respectively. Patients were grouped into quartiles of MHC-I mean expression score for each dataset. Fluctuations in patient numbers per group were caused by missing survival data. All P-values from Cox regression.

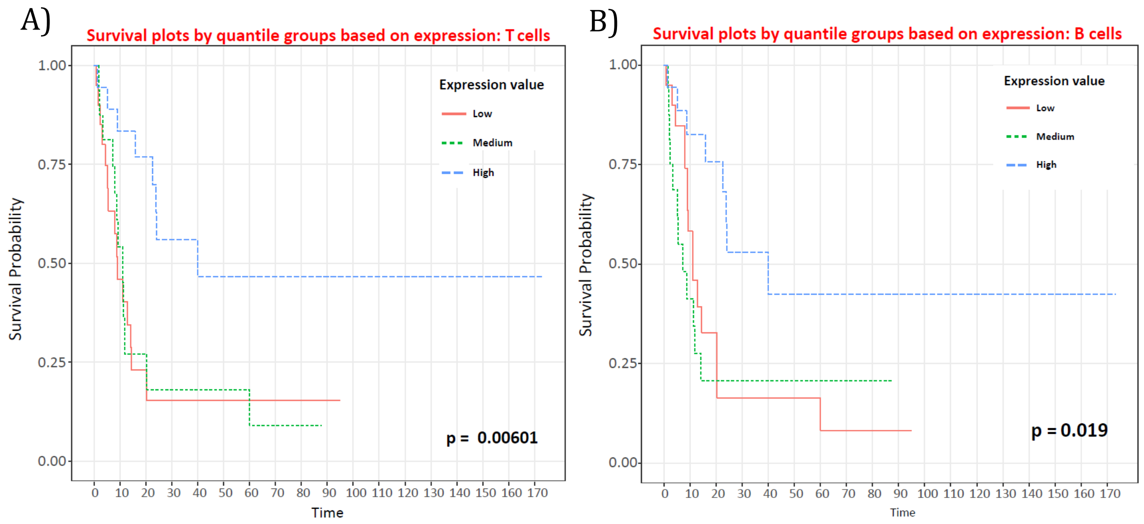

Supplementary Figure 7. Overall survival by T (**A**) and B (**B**) cell signatures as described in Tirosh et al.[6] in TCGA stage IV melanoma. All P-values from Cox regression.

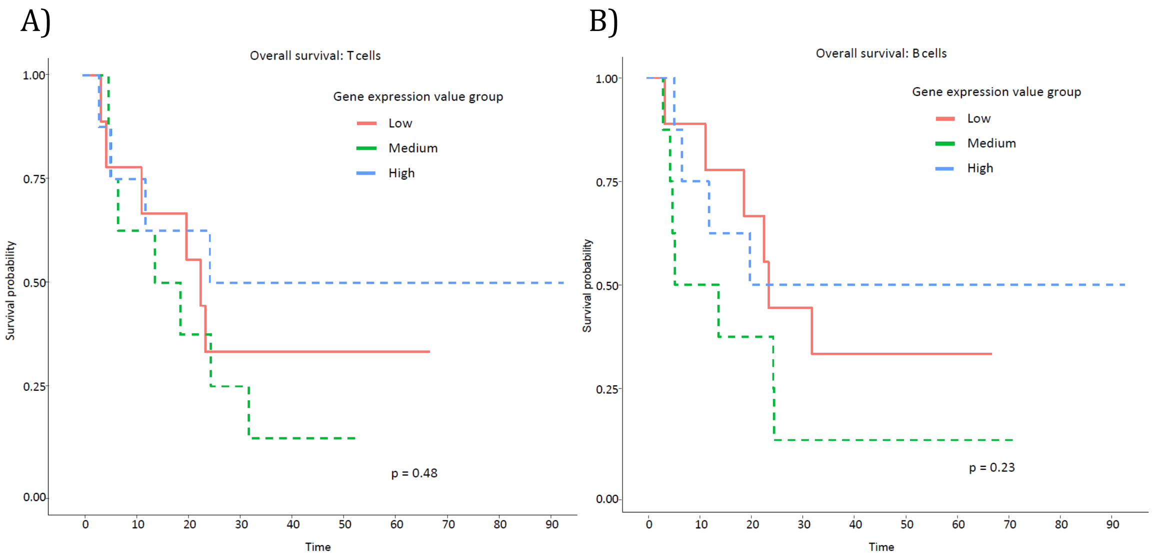

Supplementary Figure 8. Overall survival by T (**A**) and B (**B**) cell signatures as described in Tirosh et al.[6] in ACT data. All P-values from Cox regression

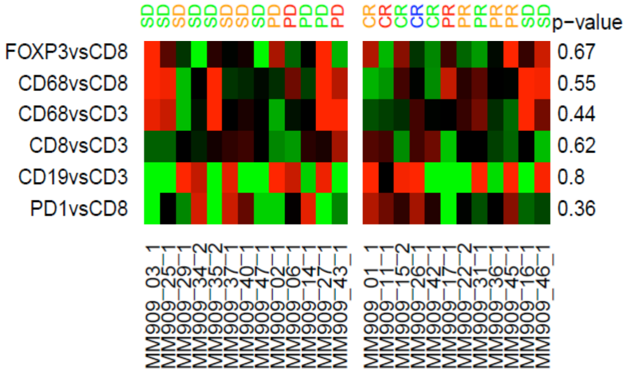

Supplementary Figure 9. Ratios of expression of immune cell markers in tumors from patients with no clinical benefit (left) and patients with clinical benefit (right) derived from RNAseq data. The P-values from t-test for test of differential expression between the groups are shown on the right.

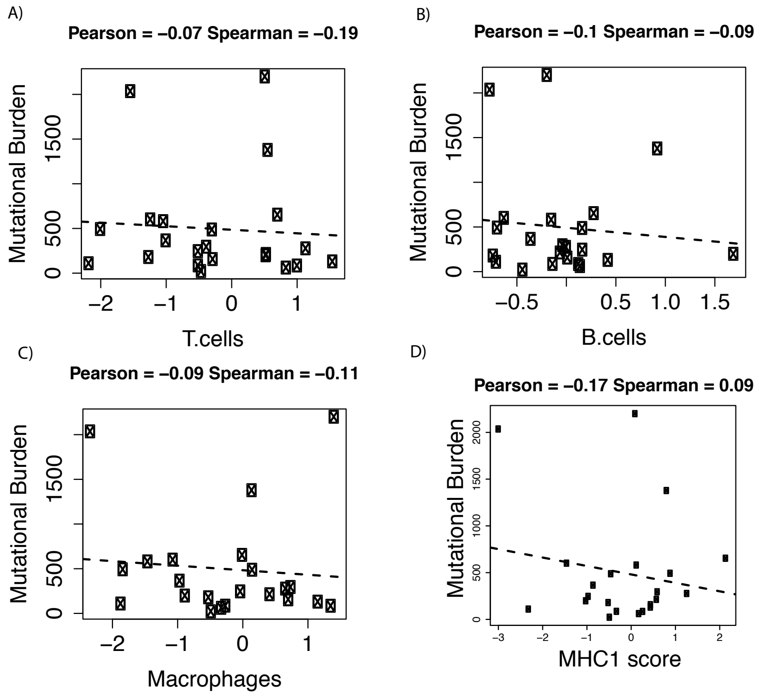

Supplementary Figure 10. Correlation of mutational burden to expression of immune cell signatures (A-C) and MHC1 score (D) in the ACT cohort. Pearson and Spearman correlation coefficient and linear regression line are indicated.

Supplementary Table 1. Gene ontology analysis and Gene Set Enrichment (GSEA) comparing tumors from patients with and without clinical benefit from ACT.

GO Term	FDR	GSEA Signature	NES	FDR
GO-term Clinical Benefit		GSEA Clinical Benefit		
IPR003597:Immunoglobulin C1-set	2,27E-09	REACTOME_IMMUNE_SYSTEM	-6,2049475	0
IPR003006:Immunoglobulin/major histocompatibility complex, conserved site	2,02E-08	REACTOME_ADAPTIVE_IMMUNE_SYSTEM	-5,1658683	0
hsa04612:Antigen processing and presentation	7,89E-08	REACTOME_CYTOKINE_SIGNALING_IN_IMMUNE_SYSTEM	-4,3279705	0
IPR011162:MHC classes I/II-like antigen recognition protein	2,94E-07	REACTOME_INTERACTIONS_OF_LYMPH._AND_NON_LYMPH._CELL	-3,6414719	0
GO:0002504~antigen proc. and pres. of pept. or polysacch. antigen via MHC II	4,26E-07	REACTOME_INTERFERON_GAMMA_SIGNALING	-3,5601988	0
IPR014745:MHC class II, alpha/beta chain, N-terminal	5,14E-07	REACTOME_INTERFERON_SIGNALING	-3,3457727	2,44E-05
hsa05320:Autoimmune thyroid disease	6,52E-07	REACTOME_TCR_SIGNALING	-3,2391462	2,12E-05
GO:0042613~MHC class II protein complex	8,46E-07	REACTOME_COSTIMULATION_BY_THE_CD28_FAMILY	-3,2355878	2,11E-05
6p21.3	6,29E-06			
hsa05330:Allograft rejection	1,16E-05	GSEA No Clinical Benefit		
GO:0032395~MHC class II receptor activity	1,33E-05	REACTOME_CELL_CYCLE	4,9598265	0
hsa05332:Graft-versus-host disease	2,28E-05	REACTOME_CHROMOSOME_MAINTENANCE	4,085007	0
hsa05310:Asthma	3,86E-05	REACTOME_CENPA_CONTAINING_NUCLEOSOMES_AT_CENTROMERE	3,9194016	0
hsa04940:Type I diabetes mellitus	4,27E-05	REACTOME_CELL_CYCLE_MITOTIC	3,897048	0
GO:0012507~ER to Golgi transport vesicle membrane	1,02E-04	REACTOME_RNA_POL_I_PROMOTER_OPENING	3,7152874	0
		REACTOME_DNA_REPLICATION	3,5948083	0
GO-term No Clincial Benefit		REACTOME_MEIOTIC_RECOMBINATION	3,5742686	0
GO:0046982~protein heterodimerization activity	2,855666301	REACTOME_MITOTIC_M_M_G1_PHASES	3,5113454	0

Supplementary references

1. Alexandrov, L.B. *et al.* Signatures of mutational processes in human cancer. *Nature* **500**, 415-21 (2013).
2. Jonsson, G. *et al.* Gene expression profiling-based identification of molecular subtypes in stage IV melanomas with different clinical outcome. *Clin Cancer Res* **16**, 3356-67 (2010).
3. Hugo, W. *et al.* Genomic and Transcriptomic Features of Response to Anti-PD-1 Therapy in Metastatic Melanoma. *Cell* **165**, 35-44 (2016).
4. Cirenajwis, H. *et al.* Molecular stratification of metastatic melanoma using gene expression profiling: Prediction of survival outcome and benefit from molecular targeted therapy. *Oncotarget* **6**, 12297-309 (2015).
5. TCGA. Genomic Classification of Cutaneous Melanoma. *Cell* **161**, 1681-96 (2015).
6. Tirosh, I. *et al.* Dissecting the multicellular ecosystem of metastatic melanoma by single-cell RNA-seq. *Science* **352**, 189-96 (2016).
