# Supplementary material for: Mutational and putative neoantigen load predict clinical benefit of adoptive T cell therapy in melanoma
Source: Nat Commun. 2017 Nov 23;8:1738. doi: 10.1038/s41467-017-01460-0 (PMC5701046; doi:10.1038/s41467-017-01460-0)
Supplement: Supplementary file 3 — Description of Additional Supplementary Files [file 41467_2017_1460_MOESM3_ESM.pdf]

Description of Supplementary Files

File Name: Supplementary Data 1
Description: Clinical annotation data

File Name: Supplementary Data 2
Description: DNA copy number data

File Name: Supplementary Data 3
Description: Somatic mutations
